# Pesticide Poisoning among All Poisoning Cases Presenting to the Emergency Department of a Tertiary Care Hospital: A Descriptive Cross-sectional Study

**DOI:** 10.31729/jnma.7131

**Published:** 2021-12-31

**Authors:** Rajesh Kumar Shah, Sidarth Timsinha, Sanjib Kumar Sah

**Affiliations:** 1 Department of Forensic Medicine, Birat Medical College Teaching Hospital, Budhiganga-2 Tanki, Morang, Nepal; 2 Department of Anatomy, Birat Medical College Teaching Hospital, Budhiganga-2 Tanki, Morang, Nepal

**Keywords:** *emergency*, *organophosphates*, *poisoning*, *suicide*

## Abstract

**Introduction:**

Acute pesticide poisoning is a significant global public health issue that contributes to one of the leading causes of emergency department visits. There is no national data on the incidence of acute pesticide poisoning or the pesticides that cause deaths. The purpose of this study is to find the prevalence of pesticide poisoning among patients who presented to the emergency department with acute poisoning.

**Methods:**

This was a descriptive cross-sectional study undertaken in a tertiary care hospital from April to September 2021 among patients who presented to the emergency department with acute poisoning. Ethical clearance was obtained from (reference number: 123/2077-78). Convenient sampling was done. Sociodemographic factors, types of poison consumed, route of consumption, reason, motive, and place of poison intake, time elapse in the presentation to the hospital were studied along with psychological factors associated with poisoning. Statistical analysis was done using Statistical Package for the Social Sciences version 23. Point estimate at 90% Confidence Interval was calculated along with frequency and proportion for binary data.

**Results:**

Out of 85 cases studied, the prevalence of pesticide poisoning was 60 (70.58%) (61.28-79.88 at 90% Confidence Interval). Insecticides 41 (68.33%) was mainly responsible for poisoning with organophosphate compounds 33 (42.30%), being the commonest chemical constituent. Fifty-three (88.33 %) incidents occurred at home. Domestic disputes 26 (43.33%) were the main reason behind poison consumption and suicide 43 (71.66%) was the main motive.

**Conclusions:**

The prevalence of pesticide poisoning among all cases of poisoning presenting to the emergency department was slightly higher than studies done earlier in similar settings.

## INTRODUCTION

Acute pesticide poisoning is a major medical emergency that causes significant morbidity and mortality in a developing country like Nepal.^1^ Pesticide poisoning is responsible for 14-20% of global suicides, and approximately 110,000-168,000 deaths each year.^2^

In Nepal, there is no national data on the incidence of acute pesticide poisoning or the pesticides that cause deaths.^3^ Record-keeping at hospitals is also under-resourced limiting its usefulness for analysis of the precise poisons involved in patients and deaths.^4^

The epidemiological factors like geographical location, occupation, socioeconomic status, literacy rate, and cultural and religious traditions can significantly influence the clinical presentation and outcome of poisoning patients. This highlights the significance of ongoing research to better understand the pattern of poisoning in a certain geographical area.^5^

Thus, the study intends to find out the prevalence of acute pesticide poisoning among patients who presented to a tertiary care hospital of Eastern Nepal.

## METHODS

This descriptive cross-sectional study was conducted at Birat Medical College Teaching Hospital (BMCTH) Tankisinuwari, Morang from April 30 to September 30, 2021. Ethical clearance was obtained from the Birat Medical College Teaching Hospital Institutional Review Committee (reference number: 123/2077-78.) All acute poisoning patients presenting to the Emergency Department were studied. All acute poisoning patients irrespective of age, sex, type and manner of poisoning and the outcome of patients were included in the study. Those who were exposed to either household or agricultural pesticides irrespective of presence of signs and symptoms, accompanied or unaccompanied by container or poison were studied. Patients presenting with history of stings bite, snake bite, industrial toxins, toxic plants, drug, or miscellaneous products were excluded. Convenient sampling was done and the sample size was calculated using the formula as,

n = Z^2^ × p × q / e^2^

  = (1.645)^2^ × (0.5) × (1-0.5) / (0.08)^2^

  = 84

Where,

n= required sample size
Z= 1.645 at 90% Confidence Interval (CI)
p= prevalence taken as 50% for maximum sample size
q= 1-p
e= margin of error, 8%

The minimum sample size was 84. However, 85 patients were enrolled after obtaining written consent. Epidemiological factors like age, gender, ethnicity, educational status, occupation and marital status were studied. Reason, motive and place of poison intake, time elapse in the presentation to the hospital were also considered. The types of poison consumed its form and route of administration were noted along with the psychological factors associated with poisoning. The collected data was analyzed in the Statistical Package of the Social Sciences version 23. The descriptive statistics were presented. Point estimate at 90% Confidence Interval was calculated along with frequency and proportion for binary data.

## RESULTS

A total number of 85 poisoning cases reported to the emergency department during the study period, out of which 60 (70.58%) (61.28-79.88 at 90% Confidence Interval) cases were of acute pesticide poisoning.

The age ranged between 2 to 70years with the mean age of 27.25±13.18years. The majority 44 (73.33%) of poisoning occurred over the age of 19 years. Females 38 (63.33%) outnumbered males 22 (33.66%) in pesticide poisoning with a male to female ratio of 1:1.7. The prevalence of poisoning was high in rural areas 31 (51.66%) and among females 39 (65%) in both married and unmarried categories. Higher rates of poisoning were seen in Tarai Madhesi ethnic groups 21 (35%) and Adivasis/Janajatis 20 (33.33%) ethnic groups. Most of the patients were unemployed 24 (40%) and farmers 21 (35%) by occupation. Majority of them were uneducated 32 (53.33%) and had low socioeconomic background 39 (65%) (Table 1).

**Table 1:** Sociodemographic findings of patients.

Category	n (%)
**Age Group**	
Children (upto 14years)	5 (8.33)
Adolescent (15-19years)	11 (18.33)
Adult (>19years)	44 (73.33)
**Gender**	
Male	22 (36.66)
Female	38 (63.33)
**Residence**	
Rural	31 (51.66)
Urban	29 (48.33)
**Marital status**	
Married	42 (70)
Unmarried	18 (30)
**Ethnicity**	
Brahmin/Chettri	9 (15.0)
Tarai Madhesi	21(35.0)
Dalit	7(11.66)
Adivasis/Janajatis	20 (33.33)
Others	3 (5)
**Economic status**	
Low	39 (65)
Middle	14 (23.33)
High	7 (11.66)
**Education**	
Literate	28 (46.66)
Illiterate	32 (53.33)
**Occupation**	
Unemployed	24 (40)
Farmer	21 (35)
Student	8 (13.33)
Others	7 (11.66)

All the patients had consumed poison orally and mainly in liquid 35 (58.33%) form. In 5 (8.33%) patients, alcohol was taken along with poison. The majority of poisoning incidents, 53 (88.33%) occurred at home, with 7 (11.66%) occurring in fields. Pesticides that caused poisoning were predominantly insecticides 41 (68.33%) and rodenticides 15 (25%). Herbicides 2 (3.33%) accounted for only a small proportion of the poisoning cases. The common constituent chemicals were: organophosphate compounds (OPCs) 33 (42.30%), followed by pyrethroids 27 (34.61%) and phosphides 15 (19.23%) (Table 2).

**Table 2:** Types of pesticide poisoning.

Categories	n (%)
**Type of pesticide**	
Insecticide	41 (68.33)
Rodenticide	15 (25)
Herbicide	2 (3.33)
Fungicide	0 (0)
Unknown	2 (3.33)
**Total**	60 (100)
Chemical types	
Organophosphorus	33 (42.30)
Pyrethroid	27 (34.61)
Phosphide (Zinc and Aluminium)	15 (19.23)
Unknown chemical	3 (3.84)
Dinitrophenol derivative	0 (0)
Carbamate/thiocarbamate	0 (0)
Organochlorine	0 (0)
**Total**	78 (100)

The poisoning occurred mainly during the late hours23 (38.33%) of the day i.e. (18.0124.00). The timeelapsed between poison intake and start of treatment,varied from 30minutes to 48hours. The majority 33(55%) of the patients reached thehospital within 3hours of poison intake with a mean time interval of 2:46hours. As per POP scale in majority of the patientsthe poisoning was mild 41 (68.33%) in severity. Deathfollowing consumption of poison was noted only in onecase 1 (1.66%) (Table 3).

**Table 3:** Chronological findings.

Category	n (%)
**Time of exposure**	
Morning (6:00-12:00)	16 (26.66)
Day time (12.01-18.00)	19 (31.66)
Evening (18.01-24.00)	23 (38.33)
After midnight (0.01-5.59)	2 (3.33)
**Time Lag**	
<1hour	13 (21.66)
1-3hour	33 (55)
>3hour	14 (20.33)
**Treatment in ICU**	
Yes	53 (88.33)
No	7 (11.66)
**Effects**	
Local	23 (21.29)
Systemic	50 (46.29)
Both	34 (31.48)
**Severity (POP scale)**	
Mild (0-3)	41 (68.33)
Moderate (4-7)	18 (30)
Severe (8-11)	1 (1.66)
**Hospitalization**	
Yes	53 (88.33)
No	7 (11.66)
**Total Days of Hospital stay**	
≤3 days	9 (15)
3-7 days	35 (58.33)
>7 days	16 (26.66)
**Outcome**	
Recovered	33 (55)
Expired	1 (1.66)
Referred	7 (11.66)
DOPR	6 (10)
LAMA	13 (21.66)

**Figure 1:**
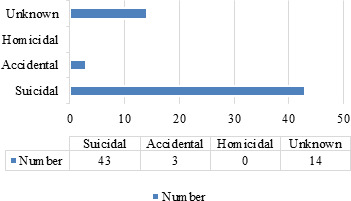
Manner of intake of poison.

In most of the patients, suicide 43 (71.66%) was the main manner of poisoning and domestic dispute 26 (43.33%) followed by financial problems 10 (16.66%) was the commonest reason of poisoning (Figure 1, Table 4). In this study only a minimum 4 (6.6%) number of victims suffered psychiatric disorder prior to poisoning. Maximum 33 (55.0%) victims survived with prompt and appropriate treatment with the mean hospital stay of 6.28days.

**Table 4:** Reason of poisoning.

Category	n (%)
Financial problems	10 (16.66)
Domestic dispute	26 (43.33)
Unsuccessful love affairs	3 (5)
Marital disharmony	7 (11.66)
Domestic violence	6 (10)
Dowry problem	2 (3.33)
Mental illness	5 (8.33)
Loss of job/employment	1 (1.66)
Total	60 (100)

## DISCUSSION

The study, like earlier studies conducted in Nepal, revealed that acute pesticide poisonings have a significant impact on young individuals between 15-30 years.^6,7^ This age group typically experience psycho-emotional issues such as academic failure, unemployment, economic hardship, unsuccessful love affairs, domestic pressure, and so on. Such issues give individuals a negative outlook towards life and are positively associated with suicidal attempts.^8^

Our study showed female predominance in poisonings, which was similar to the other studies done in Nepal.^6,7^ Domestic violence, marital relationships, and unfavorable socio-cultural norms make women more vulnerable to suicide attempts.^8^ Furthermore, females are more likely to indulge in impulsive self-harm.^9^

Pesticide poisoning was more common among housewives 35.0% of rural domicile 51.66%. Higher rural suicidal rates have been reported earlier in several Asian countries.^10,11^ The rates of poisoning were higher among Tarai madhesi ethnic groups. The majority of the victims were illiterate, and farmers with low socioeconomic background. The current finding is similar to a Nepalese study.^7^ In our study all poisoning incidences occurred at home, and mainly during the late hours of the day i.e. (6 p.m.-12:00) 38.33%. People are lonelier at this time, and inclined to recollect their problems more deeply and again, intensifying their stressful situations and leading to unfair acts. This finding is compatible with a Nepalese study^12^ but contradictory to an Indian study^13^ wherein poison was consumed mostly in the afternoon hours i.e. (noon to 6 p.m.) 43.92%.

In all of the patients, poisons was ingested orally and mainly in liquid form 58.33%. The route and form of poison intake plays a vital role in the prognosis of the case and its management. Our study finding is consistent with the result of a study.^14^ In this study the most common pesticide used was Organophosphorus compounds (OPCs) compounds in the form of chlorpyriphos plus cypermethrin. This findingis similar to a Nepalese study ^15^ but contrary to another studyconducted in Nepal which reported Metacid (Methyl-parathion) and Nuvan (Dichlorovos) as most commonly used OPCs.^16^ This disparity could be attributed to the availability and type of OPCs currently in use.

The main reason behind poisoning was domestic dispute and financial crisis. This can be directly related to issues such as poverty, illiteracy, and a variety of other stress-related factors. Similar findings were reported in a Nepalese study.^17^ Psychiatric illness didn't contribute much in poisoning in our study as there were only 4 patients out of 60 patients. However, psychiatric counselling and management was done in these patientsfollowing medical treatment. Psychiatric diseases like depressionhave been linked to high suicidal attempts in other nations.^18,19^ Evidence also suggests that in Low Middle Income Countries (LMICs), people who engage in suicidal conduct have a lower prevalence of psychiatric problems. It could be a reflection of the suicide methods used in LMICs (e.g. pesticide poisoning), which are generally impulsive acts with little or no consequences.^20^

In this study majority 68.33% of the patients had mild poisoning This could be due to less quantity of poison consumed by the victims i.e.<40ml and due to prompt hospitalization and proper management. Our finding is in accordance with a Nepali study where majority 70%of the patient had mild poisoning.^17^ However, another Nepali study reported moderate poisoning 67.80% in majority of the patients.^15^

In our study most of the patients 55% reported to the hospital within 3 hours of poison intake with a mean time interval of 2:46 hours. Study finding is in agreement with a Nepalese study.^12^ However, in one Nepalese study maximum patients reported after6 hours.^15^ Almost, all of the patients had been exposed to pesticides in their own homes, and majority of them had intentionally poisoned themselves in an attempt to commit suicide. The easy availability, extensive use and low cost of the pesticides, altogether make the population more vulnerable for suicidal as well as accidental poisoning. Study finding is in support with the findings of other workers.^12,21^

In this study, the majority 55% of patients survived, with most 58.33% patients staying in the hospital for 3-7 days. The high survival rate in our study could be due to less quantity of poison consumed by the victims, early admission and prompt and effective treatment in the hospital. Study finding is in agreement with a study conducted in Nepal.^21^

As this study was limited to a single hospital, the findings cannot be extrapolated to the entire nation. As a result, a population-based investigation is required to uncover the true extent of pesticide exposure and intoxication.

## CONCLUSIONS

The prevalence of pesticide poisoning among all cases of poisoning presenting to the emergency department was slightly higher than studies done earlier in similar settings. The most common pesticides that cause poisoning are insecticides (organophosphates and pyrethroids) and rodenticides (zinc phosphides). Pesticides are intentionally misused as an easy means to commit suicide. Pesticide poisoning is more prevalent in young people and women from low socioeconomic backgrounds.
